# An overview of spider accidents in the Brazilian Amazon

**DOI:** 10.1590/1678-9199-JVATITD-2024-0057

**Published:** 2025-04-11

**Authors:** Jonas Gama Martins, Pedro Pereira de Oliveira Pardal, Antonio Domingos Brescovit, Rudi Emerson de Lima Procópio

**Affiliations:** 1 Graduate Program in Genetics, Conservation and Evolutionary Biology, National Institute of Amazonian Research (INPA), Manaus, AM, Brazil.; 2 Laboratory of Medical Entomology and Venomous Animals, Center for Tropical Medicine, Federal University of Pará (UFPA), Belém, PA, Brazil.; 3 Laboratory of Zoological Collections, Butantan Institute, São Paulo, SP, Brazil.; 4 Graduate Program in Biotechnology and Natural Resources of the Amazon, Amazonas State University (UEA), Manaus, AM, Brazil.

**Keywords:** Amazon, Spider, Spider bite, Antivenom, Public health

## Abstract

**Background:**

Spiders of medical importance in the Amazon region belong to the genera *Phoneutria*, *Loxosceles* and *Latrodectus*. Natural history data show that *Phoneutria* spp. occur in both periodically flooded forest areas (*igapós*) and non-flooded areas (*terra firme*), as well as in commercial plantations in the Amazon. Negative interactions with wandering spiders (*Phoneutria* spp.) can occur along forest trails, leading to homes, schools and workplaces. Harmful species, such as *Loxosceles amazonica* and *Latrodectus* aff. *curacaviensis*, are mainly associated with accidents in rural settings.

**Methods:**

To understand the dynamics of spider accidents in the Brazilian Amazon, we conducted a search for scientific articles in five databases (Google Scholar, PubMed/MEDLINE, Scopus by Elsevier and SciELO). In addition, we analyzed the content of four reference books on the ecological aspects of Amazonian spiders. All told, we identified 64 eligible studies, including six regional surveys published between 1996 and 2016.

**Results:**

From 2015 to 2022, a total of 25 human lives were lost to spider envenomation in the Brazilian Amazon. An analysis of the data revealed that many riverside families engage in agricultural practices that expose them to venomous animals. Hospital data reveal that most patients bitten by spiders come from impoverished rural communities, which rely on public hospitals of Brazil’s Unified Health System (SUS) for medical treatment. The results indicate that spider bites in the Amazon represent a neglected public health problem, especially in locations far from capital cities.

**Conclusion:**

Amerindian and non-Amerindian communities living in areas at high risk of venomous animal attacks do not receive adequate attention in health policies. Given the wide dispersion of rural populations vulnerable to venomous animal incidents in the Amazon, the establishment of new referral medical centers is an essential strategy, especially for riverside communities with limited access to health services.

## Background

The World Spider Catalogue lists 52,082 described species (updated on May 22, 2024) [1]. In the Amazon Basin, it is estimated that there are 4,000 to 8,000 species of spiders [2]. Although spiders are perceived as scary and dangerous creatures by many people, the number of spider species that are potentially harmful to humans is insignificant compared to the diversity of the Order Araneae [3-5]. The largest spiders in the world (*Theraphosa* spp.), popularly known as ‘Goliath spiders’ or ‘bird eaters’, live on the Amazon and do not pose any health risk to residents, both Amerindians and non-Amerindians.

In Brazil, most states are home to spider species feared for the effects of their venom [3, 5]. These animals are responsible for accidents ranging from mild to severe symptoms, often requiring treatment with specific antivenoms [6-9]. The main species causing envenomation with clinical repercussions in urban and rural areas belong to the genera *Phoneutria* Perty, 1833, *Loxosceles* Heineken and Lowe, 1832 and *Latrodectus* Walckenaer, 1805 [9, 10].

The venom of the brown recluse spider (*Loxosceles* spp.) causes local pain and progressive necrotic lesions [11, 12]. The bite of the wandering spider (*Phoneutria* spp.) can trigger mild, moderate or severe symptoms [10, 13]. Although there is no record of national production of therapeutic antibodies against the toxins of the widow spider (*Latrodectus* spp.) [10], moderate and severe envenomation by this spider requires parenteral administration of antivenom.

The South and Southeast regions of Brazil record the highest average annual number of accidents involving spiders [9, 10]. However, the highest fatality rates are recorded in the North of the country [10]. In 2021, the highest fatality rate due to spider envenomation occurred in the state of Roraima (2°54'29.5"N 60°53'26.4"W), located in the extreme north of the country [10].

In rural Amazon, both indigenous and non-indigenous people face risks of interacting with potentially harmful spiders, many of which still lack studies [13, 14]. Subsistence activities such as hunting, fishing and fruit gathering expose Amerindian communities to a greater risk of accidents involving venomous animals [15]. Rural communities of rubber tappers, indigenous people and hunters, who often work with bare hands and feet, are among the groups most vulnerable to attacks by venomous animals. The low light on forest trails, due to the closed canopy, reduces the perception of spiders hidden in the soil and vegetation. The use of flashlights and closed footwear is recommended to mitigate these risks [16].

In the northwest of the Amazon region, spider-related accidents are often ignored by epidemiological services [13, 17]. In remote areas, there is a high probability that cases will not be reported to official health agencies [18]. Amazonian spiders can cause symptoms ranging from mild to severe, and surgical intervention is necessary in cases of compartment syndrome resulting from accidents with spiders of the genus *Phoneutria* spp. [13, 14].

The separation of spider populations by large rivers in the region can result in peculiar symptoms in patients [5, 13, 18], possibly due to variations in the composition of animal venom. However, there is still a lack of updated epidemiological data on spider envenomation in the Brazilian Amazon, including the identification of the main causes of accidents, risk groups, and perceptions of rural communities regarding this type of animal envenomation.

The aim of this study is to describe the epidemiology of spider-related accidents in the Brazilian Amazon between 1996 and 2016, using data from the Notifiable Diseases Information System (SINAN). To estimate the frequency of antivenom use in the analyzed period, we considered data from the Strategic Inputs Information System (SIES), a platform linked to the Brazilian Ministry of Health that consolidates information from state health departments on the allocation and use of immunobiologics. By recording detailed data on the distribution and use of antivenoms, these data allow an analysis of the demands on the health systems in states affected by accidents involving venomous animals.

The analysis of epidemiological data from SINAN includes estimating the average annual number of accidents, incidence and fatality rates, as well as the monthly distribution of accidents in the states of the Brazilian Amazon. We also examine the risk factors associated with negative interactions with spiders, highlighting the importance of preventive measures and management strategies to reduce the impacts of accidents on the health of communities vulnerable to venomous animals.

## Methods

The scientific information available in the literature was retrieved from Google Scholar, PubMed/MEDLINE, Scopus (Elsevier), PubMed, and SciELO databases. In the virtual search, the used descriptors were “Brazilian Amazon,” “Amazonian spiders,” “*Phoneutria*,” “*Loxosceles*,” “*Latrodectus*,” “spider bite,” and “antivenom” without year restrictions. The epidemiology of accidents, allocation, and use of antivenoms were compiled from the Notifiable Diseases Information System (SINAN) and the Strategic Inputs Information System (SIES), respectively.

The screening of scientific manuscripts was carried out in two stages. In the first, the titles and abstracts of interest to the research were selected. Manuscripts that did not meet the established criteria were removed, and eligible studies were preselected by two reviewers. In the second stage, complete reviews of the remaining publications were performed. We selected information such as clinically important spiders, antivenom, spider bites, symptoms, risk factors, hospital admission, referral hospital, traditional treatment, distance between the accident and hospital, and target communities of venomous animals. Discrepant data and unclear conclusions were resolved by consulting a second reviewer.

**Figure 1.**
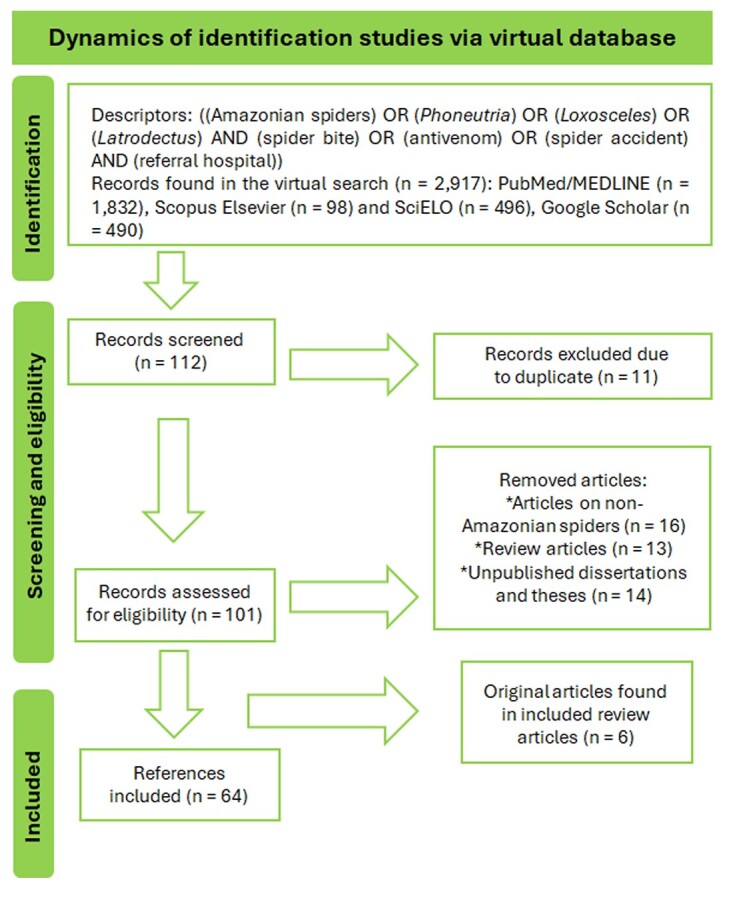
Dynamics of searching, excluding and eligibility of the references.

To deepen knowledge about spider accidents, we explored national and international physical books. Figure 1 summarizes the dynamics of searching, excluding, and the eligibility of the references that served to summarize this study. This review brings together clinical and epidemiological information and a real-world history of allocation and use of commercial antidotes against spider envenomation in the Brazilian Amazon.

## Results

### Phoneutria Perty, 1833 (Araneae: Ctenidae)

The genus *Phoneutria* groups the largest spiders of medical importance in South America [1]. Wandering spiders found in nature can have a total length of 48 mm with stretched legs [4]. The Brazilian Amazon is home to three species of *Phoneutria* Perty (1833), all described in the 19^th^ century: *Phoneutria boliviensis* F. O. Pickard-Cambridge, 1897 (states of Acre and Amazonas); *Phoneutria reidyi* F. O. Pickard-Cambridge, 1897 (states of Amapá, Amazonas, Pará and Rondônia); and *Phoneutria fera* Perty, 1833 (states of Acre, Amazonas, Pará and Roraima) [5].

**Figure 2.**
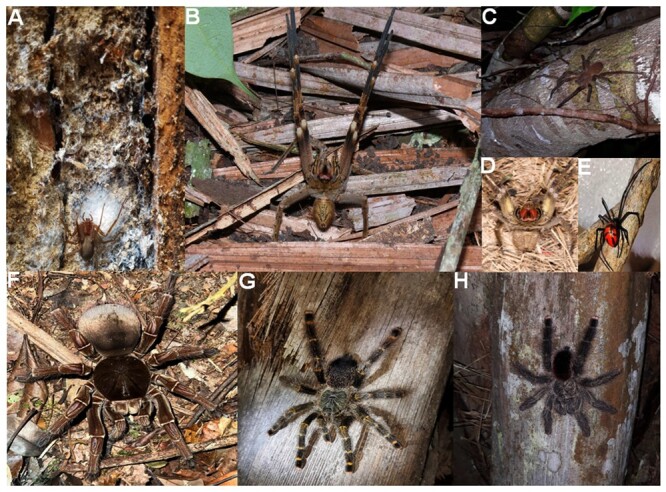
Spider species of the Brazilian Amazon. **(A)** *Loxosceles amazonica* from Belterra (2°38'17.6"S 54°56'07.9"W), Pará State. **(B)** *Phoneutria reidyi* from Barcelos (0°58'20.3"S 62°55'26.4"W), Amazonas State. **(C)** *Phoneutria fera* from Iranduba (3°06'55.0"S 60°26'21.6"W), Amazonas State. **(D)** *Phoneutria reidyi* from Belterra (2°38'17.6"S 54°56' 07.9"W), Pará State. **(E)** *Latrodectus* aff*. curacaviensis* from Manaus (3°07'14.7"S 60°00'39.8"W), Amazonas State. **(F)** *Theraphosa stirmi* from Manaus (2°57'47.1"S 59°55'22.0"W), Amazonas State. **(G)** *Avicularia rufa* from Barcelos (1°39'39.0"S 63°20'42.2"W), Amazonas State. **(H)** *Avicularia avicularia* from Iranduba (3°16'37.6"S 60°11'29.8"W), Amazonas State. Photos by **(A)** G. Porto, **(B)** F. Xavier, **(C, G, H)** J. Martins, **(D)** R. Sawaya, **(E)** J. Prestes, and **(F)** Santos. Images kindly provided by their authors and published with permission.

In the Amazon rainforest, wandering spiders are found in economically important palm trees [19]. From palm trees, rural communities extract fruits, palm hearts, oil and leaves to cover their houses [20]. The wandering spiders’ natural color and their ability to hide in different substrates in the Amazon jungle make it difficult to collect specimens for study. Wood logs harvested in forests to build houses and boats should be inspected before being handled. *Phoneutria fera*, *P. boliviensis*, and *P. reidyi* are often not detected on tree trunks by rubber tappers and hunters (Figure 2 C ). Limbs such as unprotected hands and feet are targets for spider bites.

*Phoneutria* spiders are capable of intimidating humans and natural enemies through aggressive behavior. They raise their front legs, sit on their abdomen and hind legs, display their chelicerae through which they inject their neurotoxic venom, and can jump towards attackers (Figures 2 B  -2D). Male and female wandering spiders paralyze their prey by injecting venom through a pair of chelicerae that vary in length from 4.2 to 4.8 mm [21].

In the immense Amazon basin, populations of wandering spiders are separated by large rivers and tropical forest stands [5, 18, 19, 22]. Scientific expeditions in the Amazon found *P. reidyi* (Figure 2 D ) close to tributaries of white water (Purus River) and black water (Negro River) watercourses [5]. Our photographic record documents a specimen of *P. fera* (Figure 2 C ) in a seasonally flooded forest (*igapó*) in the municipality of Iranduba (3°06'55.0"S, 60°26'21.6"W), Manaus, Amazonas, Brazil.

**Figure 3.**
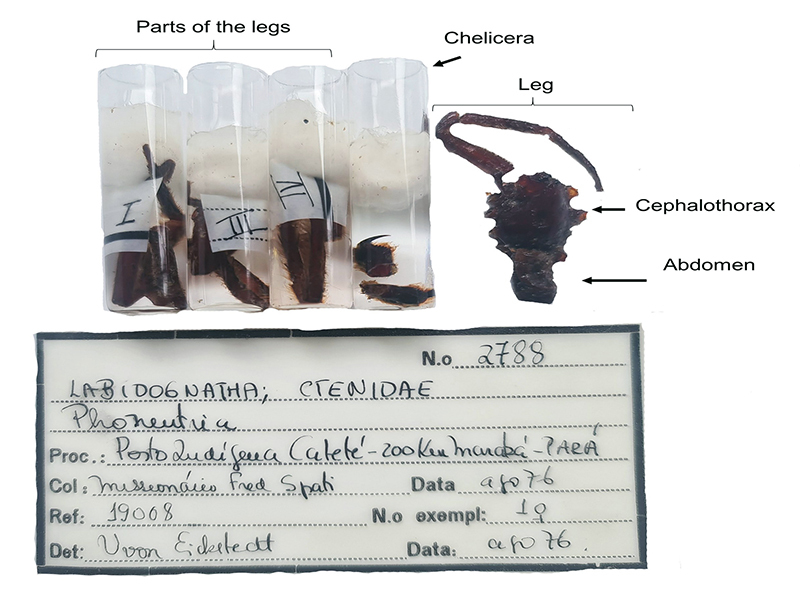
A female *Phoneutria reidyi* found in the Xikrin indigenous community (6°14'03.1"S 50°48'27.1"W), Pará State, Brazil. The animal was sent by a missionary, Fred Spati, to the Butantan Institute in 1976 after a series of envenomings in the community. Photo by A. Brescovit.

In the Amazon region, there are few studies on spider envenomation in indigenous territories [9, 10, 22]. To prove that wandering spiders were responsible for the envenomation in the Xikrin Indigenous Community (6°14'03.1"S 50°48'27.1"W), in Pará, a missionary who lived with the natives sent a specimen to the Butantan Institute in 1971 [18]. The spider was identified as a female *P. reidyi* (Figure 3 D) that had bitten Amerindian adults and children [18]. The closest municipal referral hospital to the Xikrin village of Pará is located in Marabá (5°20'27.9"S 49°05'10.1"W), in the southeast of the state [23].

Recent natural records of *P. fera* suggest that this spider can cause accidents in rural communities in the Colombian Amazon, such as in the departments of Vaupés (0°39'19.0"N 70°38'33.1"W), Boyacá (5°27'15.6"N 73°21'50.5"W), and Putumayo (0°37'16.1"N 76°01'29.7"W) [24]. In the Colombian Amazon, a farmer bitten by *Phoneutria* sp. presented cardiovascular manifestations and compartment syndrome [13]. One milligram of atropine was administered intravenously to reduce the effects of the venom, but standard medication did not reverse the cardiovascular manifestations in the patient.

An experimental study in Brazil administered wandering spider venom to mammals and observed symptoms such as pain, sneezing, tearing, hypersalivation, ataxia, vomiting, and erection, ejaculation, and, in some cases, death [25]. Fortunately, the antivenom against *Phoneutria* spp. spider toxins produced in Brazil is efficient and neutralizes the effects of the toxins both in experimental models and in a hospital environment [8, 11, 12].

### *Loxosceles* Heineken and Lowe, 1832 (Araneae: Sicariidae)

Brown spiders (*Loxosceles* spp.) are apparently harmless (Figure 2 A ). They are small (2-3 cm long), not aggressive, and their bites do not cause stinging pain [12]. The small size and cryptic color of brown spiders make them difficult to detect in homes and in nature (Figure 2 A ) [26].

*Loxosceles amazonica* Gertsch, 1967 (Figure 2 A ) is the only brown spider recorded in the Brazilian Amazon [14, 27].*Loxosceles amazonica* occurs in the states of Roraima, Amazonas, and Pará [27]. From 2007 to 2014, a total of 156 spider accidents in Amazonas were attributed to *Loxosceles* spp. [14]. New records of the natural distribution of *L. amazonica* show that they occur mainly in rural areas [27]. 

In the Amazon region, the epidemiology of brown spider envenoming is most reported for the Peruvian Amazon, where more than 8,000 spider accidents and eight deaths were reported from 2016 to 2021 [28]. *Loxosceles laeta* is a public health problem in the Peruvian Amazon [28]. Brown spiders develop venoms with enough destructive activity to cause tissue loss in any part of the human body [29]. Dermonecrotic components injected cause necrosis that increases in diameter [30-33]. Animal venoms of this nature are definitely a risk to human health [34-37].

A potential bottleneck for the efficient treatment of loxoscelism is that many patients are unaware that they have been bitten by a brown spider [11, 12]. In the Amazon region, medical professionals who treat people bitten or stung by venomous animals must be aware of the similarity between cutaneous loxoscelism and other dermatological diseases common in the region, such as leishmaniasis [11]. Accidents caused by brown spiders should receive medical evaluation, and when necessary, commercial antivenom must be used.

Antivenom therapy counteracts the potency of the venom and reduces the progression of necrosis at the site [30-35]. Victims of loxoscelism who delay seeking medical attention may suffer injury enlargement, requiring surgery to speed up the healing process [11]. Perhaps brown spider envenoming in the Brazilian Amazon is not as common as in the Southern region of the country, but there are clinical records in the state of Amazonas of patients with brown spider-induced injuries [14].

### *Latrodectus* Walckenaer, 1805 (Araneae: Theridiidae)

Andean communities in South America call black widow spiders (*Latrodectus* spp.) “*mico mico*” or “*huayruro*” [34]. Populations of *Latrodectus* spp. in Latin America have been recorded in Colombia, Chile, Uruguay, Argentina, Guyana, Suriname, Venezuela, and Peru [35]. Black widows are feared in many parts of the world, including the Amazon region [33, 34].

In Brazil, three species are named *Latrodectus geometricus* (domestic brown widow), *Latrodectus mactans,* and *Latrodectus* aff*. curacaviensis* (Figure 2 E ) [33]. Molecular data of *Latrodectus* sp. from Brazil suggest that a taxonomic revision of the group is needed [38, 39]. Black widow venom inoculated in mammals causes massive release of neurotransmitters [39, 40]. Black widow α-latrotoxins in the human body cause abdominal pain, sweating, hypertension, and vomiting [41].

In the Amazon region, black widow spiders cause accidents mainly in regions of Bolivia. Envenomings have occurred in Tarija (21°31'02.1"S 64°43'47.0"W), Chuquisaca (18°57'58.3"S 65°16'03.5"W), Cochabamba (17°24'51.6"S 66°10'04.8"W), and La Paz (16°29'25.4"S 68°07'21.8"W) [34, 36, 39]. Bolivia produces a specific antivenom [36].

Unfortunately, a woman from a rural community in the Bolivian Amazon lost sensitivity in her lower limbs after being bitten by a black widow specimen [36]. From 2007 to 2014, 19 spider accidents in the state of Amazonas were attributed to black widows [14]. An 11-year-old boy bitten by a black widow in Manaus (capital of Amazonas) was treated at the Heitor Vieira Dourado Institute of Tropical Medicine (FMT-HVD). The patient was suffering from headache, nausea, vomiting, abdominal pain, fever, sweating, tremors, and delirium [41]. The patient received symptomatic treatment, and the effects of the envenoming were resolved.

In Latin America, research in Argentina showed that the effects of black widow venom are reversed in less time with antivenom therapy [40]. The Butantan Institute began producing an experimental antivenom against these spiders in the 1960s, but the low frequency of accidents led to the interruption of the therapeutic project [37]. The Vital Brazil Institute (22°54'20.8"S 43°05'49.5"W), located in the state of Rio de Janeiro, Southeast region of the country, registered an antidote against black widow spider toxins [35]. Brazil currently does not produce antivenom against black widow toxins [35, 37, 41].

### Estimate of accidents caused by spiders in the Brazilian Amazon

We compiled electronic records of spider accidents in the Brazilian Amazon from the Notifiable Disease Information System (SINAN). This national database is fed by state health secretariats throughout Brazil [10]. Table 1 shows the characteristics of spider envenomation in the Brazilian Amazon from 2015 to 2022. 

**Table 1.** Characteristics of the 12,126 spider accidents in the Brazilian Amazon from 2015 to 2022.

Area of occurrence (n = 11,823; 97.5%)	Number	%
Rural	6004	49.5
Urban	5664	47.1
Peri-urban	155	1.2
**Gender (n = 12,126; 100%)**		
Male	6908	56.8
Female	5218	43.0
**Work-related accident (10,647; 87.8%)**		
Yes	1691	13.8
No	8956	73.8
**Ethnicity (n = 11,607; 95.7%)**		
Asian	102	0.8
White	1708	14.1
Black	696	5.7
Mixed	8621	71.1
Indigenous	480	3.5
**Education in years (n = 7,425; 61.2%)**		
Illiterate	363	3.3
1-4	1374	11.3
5-8	1501	12.3
≥ 8	4187	34.5
**Potential type of envenomation (n = 9,467; 78.0%)**		
*Phoneutria*	1520	12.5
*Loxosceles*	1982	16.3
*Latrodectus*	93	0.7
Other genera	5872	48.4

**Figure 4.**
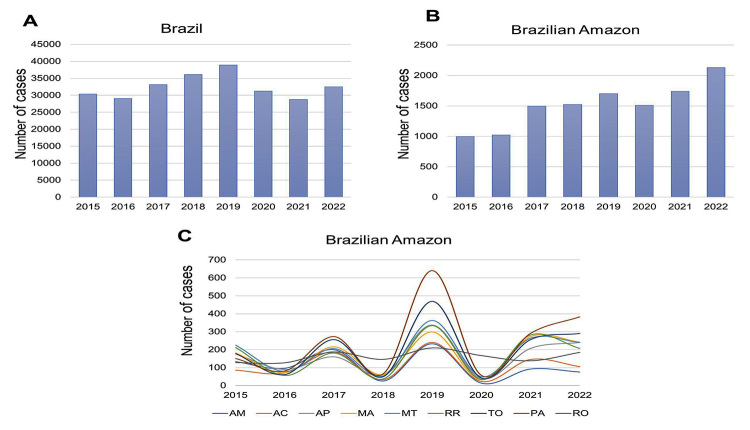
Time distribution of spider bites in Brazil, Brazilian Amazon and Amazonian states, from 2015 to 2022.

States in the Brazilian Amazon, including Maranhão (5°23'49.9"S 46°27'50.6"W) and Mato Grosso (15°36'00.6"S 56°06'18.6"W), partially covered by the Amazon rainforest, recorded 12,126 accidents caused by spiders (Figure 4), while the majority of patients envenomed by spiders were men between 20 and 60 years of age. Spider bites mainly affected the lower (49.4%) and upper (42.9%) limbs. From 2015 to 2022, spiders caused 247,047 accidents and 111 deaths (fatality rate of 0.04%) (Table 2). During this period, 25 deaths from spider envenoming were reported in the Brazilian Amazon (fatality rate of 0.20%) [OR = 4.6, (95%CI 0.18-0.49), p = 0.006] (Table 2). 

**Table 2.** Risk of lethality from spider bites in Amazonian states compared to non-Amazonian regions of Brazil.

Region	Cases	Deaths	Lethality (%)	OR (IC_95_%)	p
Rondônia	1349	2	0.14	3.31 (0.05-0.55)	0.0174803
Acre	610	-	-	-	-
Amazonas	1679	3	0.17	3.39 (0.03-0.70)	0.353123
Roraima	384	3	0.78	17.6 (0.03-0.70)	0.353123
Pará	2934	3	0.10	2.27 (0.03-0.70)	0.082245
Amapá	288	-	-	-	-
Tocantins	1811	-	-	-	-
Maranhão	1788	11	0.61	13.78 (0.39-2.34)	0.171013
Mato Grosso	1289	3	0.23	5.18 (0.03-0.70)	0.082245
Brazilian Amazon	12126	25	0.20	4.6 (0.18-0.49)	0.0062583
Non-Amazonian regions	247047	111	0.04	-	-

**Figure 5.**
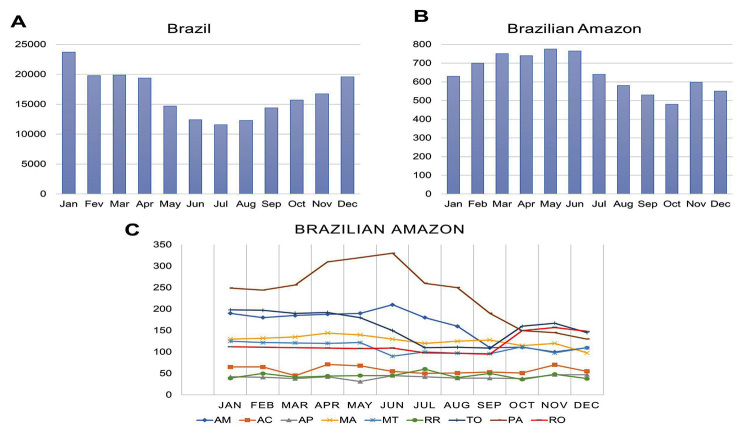
Monthly distribution of spider bites in Brazil, Brazilian Amazon and Amazonian states, from 2015 to 2022.

**Figure 6.**
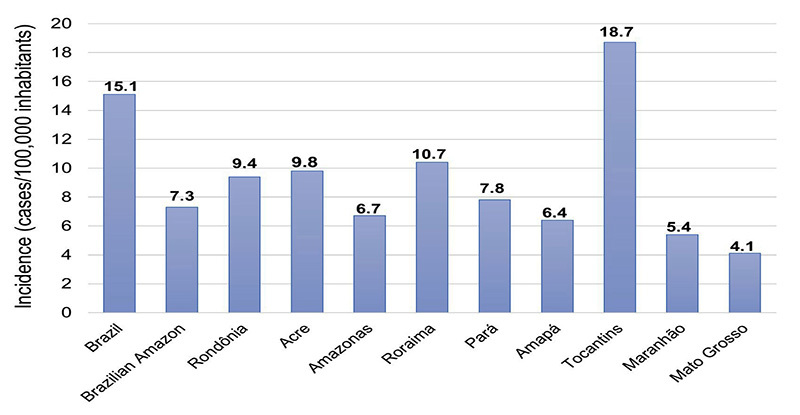
Incidence of spider bites in Brazil, Brazilian Amazon and Amazonian states in 2022.

Spider envenomation in the Amazon was most frequent from April to June (Figure 5 B ). The highest spider bite rates were detected in the states of Tocantins (18.7 cases/100,000 inhabitants), Roraima (10.4 cases/100,000 inhabitants) and Rondônia (9.4 cases/100,000 inhabitants) (Figure 6). The estimated incidence of spider bites by states in the Amazon region ranged from 4.1 to 18.7 cases/100,000 inhabitants (Figure 6). 

The estimated incidence for the state of Tocantins (Figure 6) was higher than the national average (18.7 cases/100,000 inhabitants) [10]. From 2015 to 2022, 4,307 (35.5%) people envenomed by spiders had access to a hospital in the Amazon region in the first three hours after the accident, and within 12 hours, 2,996 (24.7%) received medical care. Antivenom therapy was recommended for 2,436 patients (20.8%). 

## Discussion

The incidence rate of spider envenomation varies among states in the Amazon region (Figure 6). In Tocantins, for example, the estimated incidence rate exceeded that of the states with the highest number of deaths, namely Maranhão and Pará (Table 2). Between 2010 and 2020, 2,510 vials of antivenom were distributed in Tocantins, of which only 890 were effectively used in referral medical centers (Table 3). During this period, referral hospitals in the region treated 900 men and 911 women victims of spider envenomation. Variations in the incidence of accidents in the Brazilian Amazon are shown in Figure 6.

The highest lethality rate due to spider envenomation was observed in the state of Roraima (Table 2), located in the extreme north of the Brazilian Amazon. Species such as *P. fera*, *L. amazonica* and *L. geometricus* occur in natural sites in Roraima [27, 41]. The spider bite lethality rate in Roraima was 0.78%, while in non-Amazonian states it was 0.04% (Table 2). The dynamics of accidents in the region, as well as the circumstances in which the envenomation occurred are rarely documented. Our study detected 11 deaths from spider bites in the state of Maranhão from 2015 to 2022 (Table 2). Higher chances of lethality have been detected in Roraima, Maranhão and Mato Grosso (Table 2). Deaths from animal envenoming in the Amazon are linked to the delays in hospital treatment and the lack of regular health services in high-risk areas [14, 42]. Sustenance activities such as hunting and fishing bring people into close contact with venomous animals [15, 20].

The state of Pará, the most populous in the North, had the highest number of spider-related accidents (Figure 4 C ). Spider envenomation in Pará increased during the rainy season (April to June) and decreased during the rest of the year (Figure 5 B ). During the rainy season, rising water levels flood the habitats of venomous animals, causing them to migrate to dry areas to survive [18, 19]. The seasonal migration of venomous animals in the Amazon may increase the risk of accidents [18].

Many accidents involving venomous animals in indigenous communities in Pará are not formally reported [13]. Local native groups prefer their own remedies for symptoms of envenomation [18, 22, 42]. Although the Special Indigenous Health Districts (DSEIs) of the Brazilian Amazon regularly receive vaccines and therapeutic serum from agencies in the capitals [23]. Perhaps some tribes are unaware that there is effective treatment in referral hospitals for animal envenoming [22, 42, 43].

Data from the Strategic Inputs Information System (SIES) indicate a regular distribution of antivenoms in the Amazonian states affected by spider envenomation (Table 3). On the other hand, these data do not reveal the equitable local distribution of antivenoms. The logistics of distributing hospital supplies to remote communities faces serious challenges, aggravated by the precarious health infrastructure and the shortage of trained professionals in rural areas [28, 36].

The concentration of professionals in the capitals contributes to inequalities in regional health services [44]. Overcoming regional health inequalities in the Amazon requires strong public policy involvement to ensure the installation of new hospitals along with the production and distribution of antivenoms and adequate treatment of patients envenomated by venomous animals.

Deaths from spider envenomation in the Brazilian Amazon should be addressed by regional health authorities as an urgent need for regional clinical and epidemiological studies. Such investigations should prioritize the identification of the spider species involved, the analysis of the underlying causes of the accidents, and the assessment of access to serotherapy. In addition, educational campaigns targeting vulnerable rural communities and the training of local health professionals are essential to reduce the risks associated with envenomation by venomous animals.

### Recommendations for “*piaçabeiros*”, rubber tappers, hunters and fishers

The risks of accidents involving venomous animals in the Amazon rainforest are not the same for everyone (Table 1). Fatal encounters with spiders occur mainly in rural areas (Tables 1 and 2). Fishers, hunters and ‘*piaçabeiros’* (collectors of *piassava* - *Leopoldinia piassaba* Wallace- palm fruits and fiber) must remain vigilant while catching/collecting and transporting non-timber products (e.g., bunches of fruits, nuts, vines, seeds and palm hearts).

Arboreal spiders such as *P. fera* and *A. rufa* (Figure 2 CG ) hiding in palm trees can easily bite farm workers and extractivists. For manual work in a forest environment, riverside people need to visually inspect the surroundings to try to detect harmful animals. Sitting on fallen tree trunks requires attention. Indigenous and non-indigenous community members must increase vigilance in “controllable” environments, such as inside their homes, during river floods. The magnitude of flooding in the Amazon destroys natural shelters for venomous animals and drives their horizontal and vertical migration [15, 16, 45].

Rubber tapper communities in Vale do Juruá (7°36'49.9"S 72°36'11.9"W), Acre state, Brazil report many problems with venomous animals when handling forest products [46]. In the Amazon region, barefoot fishermen near riverbanks at night can step on wandering spiders (Figure 2 B ). These spiders are typically found outside their shelters between 7 p.m. and midnight [19, 47]. 

Night hunting of animals on the Amazon can be risky because: (i) many species of venomous animals are more active at night; (ii) forest trails are narrow and dark; and (iii) hunters' visual perception is reduced in dark environments. Any member of the human body that comes into physical contact with the wandering spider can easily be bitten. Our data show that body parts such as hands and feet (Table 1) are most commonly affected by spiders in the Amazon, suggesting that many residents of rural communities do not use personal protective equipment.

Victims of envenoming by venomous animals should seek medical assistance at the nearest hospital. Antivenom is the standard treatment to neutralize the toxins produced by venomous animals. Non-indigenous communities should not use traditional treatments for animal envenoming.

Health education programs targeting rural communities vulnerable to venomous animals are scarce in the Amazon [48]. Nurses and health technicians familiar with tropical diseases in the region could be trained to educate rural workers about the risks of contact with venomous animals and ways to minimize exposure to envenomation. Recommendations include the use of boots, flashlights and gloves by extractivist communities. In addition, it is essential for riverside communities to be informed about the availability of antivenoms in their regions, allowing them to quickly and effectively access medical care when needed.

### Reference hospitals and utilization of antivenom for spider bites in the Brazilian Amazon

**Figure 7.**
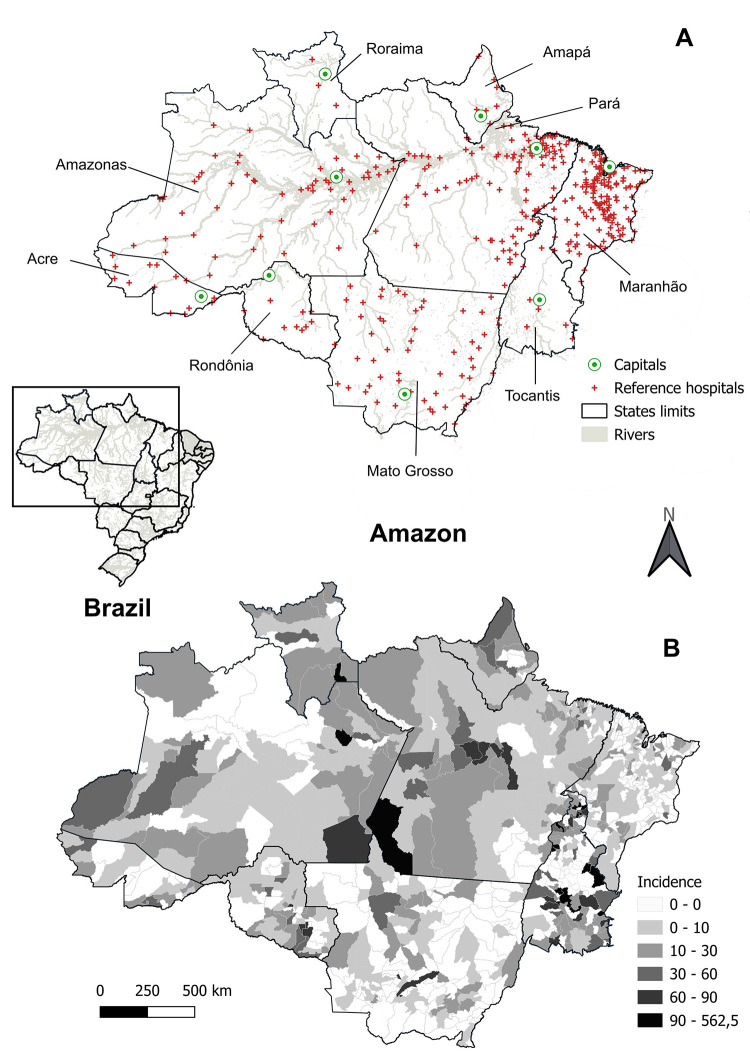
Map highlighting the Amazonian states in Brazil. **(A)** Spatial distribution of reference hospitals in the Brazilian Amazon. In the states of Amazonas, Pará and Mato Grosso, several hospitals are located close to the riverbanks, while Maranhão has a concentration of hospitals in the northern and central regions. **(B)** Incidence of spider envenomation by municipalities in the Brazilian Amazon in 2022. Points of highest incidence are observed in the center and southwest of Pará, south of Roraima and center of Tocantins. The hospital shapefile was created according to data from the Brazilian Ministry of Health (source: https://www.gov.br/saude/pt-br/assuntos/saude-de-a-a-z/a/animais-peconhentos/hospitais-de-referencia.

In the Brazilian Amazon, antivenom against spider toxins is allocated to 416 referral hospitals (Figure 7 A ) [23]. In the three largest states of the Brazilian Amazon, the number of hospitals for treating spider accidents is as follows: Amazonas, n = 58; Pará, n = 108; and Mato Grosso, n = 92. In the state of São Paulo, where the antivenom is manufactured, there are 220 hospitals for treating symptoms of accidents caused by spiders, scorpions, snakes and caterpillars [49].

To allocate antivenom batches to the states, the Brazilian Ministry of Health uses statistical methods, based on accident records in the SINAN website database [50]. The majority of antivenom batches destined for the Brazilian Amazon come from the state of São Paulo (Southeast region) and are carried by air. Since the commercial aviation service in the Brazilian Amazon is concentrated in the capitals, the allocation of antivenoms to remote municipalities is carried out by boats or small aircraft [51].

An investigation into the actual location of reference hospitals in the Brazilian Amazon showed that the majority are located in urbanized areas with better access (Figure 7 A ) [23], which in theory facilitates the acquisition of supplies by hospitals. To our knowledge, this is the first study in the Amazon region describing the spatial distribution of hospitals with antivenom stocks for spider bites.

In rural Amazon, most pediatric patients requiring intensive care are transferred to urban hospitals with better health service performance [55]. The Lower Amazonas Regional Hospital (2°27'01.6"S 54°42'53.8"W) (Santarém, Pará), for example, receives patients from distant rural communities [52, 53]. Inhabitants of riverside settlements far from urban hospitals, such as the community of Moura (1°27'22.8"S 61°38'12.9"W), in the Negro River region of Amazonas, must travel several hours by boat to Barcelos General Hospital (0°58'19.5"S 62°55'24.7"W). Residents of remote communities are most affected by the geographical distance from hospitals. The current distribution of hospitals in the region mainly favors urban populations.

In the Brazilian Amazon, medical centers specialized in the treatment of accidents caused by venomous animals, such as João de Barros Barreto University Hospital (1°27'32.2"S 48°29'37.8"W) (Belém, Pará) and the Heitor Vieira Dourado Tropical Medicine Foundation (3°07'02.4"S 60°00'41.9"W) (Manaus, Amazonas), are located in the state capitals. 

In the Middle Rio Negro region of the state of Amazonas, accidents caused by venomous animals are treated at Barcelos General Hospital (0°58'55.5"S 62°56'41.6"W). Its location approximately 200 meters from the riverbank assures accessibility to riverside communities living far from the municipality.

We compiled data from SIES and created a real historic of distribution and utilization of anti-arachnid serum in the Brazilian Amazon from 2010 to 2020 (Table 3). In 10 years, 40,378 ampoules of antivenom were allocated to the nine states of the Brazilian Amazon to treat accidents involving spiders (Table 3). All told, 14,886 ampoules of antivenom against spider toxins were administered in the Brazilian Amazon (Table 3).

**Table 3.** Allocation and utilization of antivenom for spider accidents (*Phoneutria*, *Loxosceles* and *Tityus*) in Amazonian states from 2010 to 2020.

Year	Amazonas	Acre	Amapá	Maranhão	Mato Grosso	Pará	Roraima	Rondônia	Tocantins
2010	900	110	370	430	850	3200	200	470	220
	*173*	*8*	*24*	*79*	*258*	*934*	*10*	*74*	*12*
2011	620	210	250	570	600	800	196	500	150
	*204*	*31*	*36*	*101*	*201*	*746*	*28*	*69*	*28*
2012	700	160	190	270	650	550	20	225	120
	*232*	*38*	*16*	*83*	*259*	*794*	*13*	*138*	*72*
2013	160	75	60	170	220	195	120	140	145
	*185*	*36*	*53*	*143*	*291*	*481*	*19*	*79*	*48*
2014	310	250	125	510	260	265	155	260	125
	*56*	*49*	*19*	*52*	*75*	*123*	*4*	*81*	*93*
2015	410	150	110	150	300	270	120	220	150
	*104*	*53*	*26*	*67*	*123*	*160*	*7*	*92*	*52*
2016	440	190	230	500	520	920	70	240	210
	*141*	*19*	*53*	*134*	*80*	*169*	*33*	*80*	*49*
2017	560	130	285	520	410	540	100	290	245
	*180*	*64*	*160*	*427*	*175*	*491*	*10*	*115*	*95*
2018	570	265	505	935	460	545	93	265	287
	*281*	*54*	*198*	*277*	*153*	*299*	*36*	*128*	*124*
2019	705	260	460	1340	750	960	313	530	513
	*243*	*66*	*228*	*347*	*218*	*544*	*25*	*207*	*179*
2020	515	300	365	810	430	705	110	370	345
	*231*	*48*	*183*	*302*	*179*	*298*	*8*	*182*	*138*
Total	5890	2100	2950	6205	5450	8950	1497	4755	2510
	*2030*	*466*	*996*	*2012*	*2015*	*5039*	*193*	*1245*	*890*

Source: Strategic Product Information System (SIES) and Information System for Notifiable Diseases (SINAN), updated in May 2023. 000: Underlined numbers: allocation of spider antivenom; *italicized numbers*: utilization of spider antivenom

Although there are spiders of medical importance in all states of the Brazilian Amazon, Pará was the one that most often used antivenom therapy against spider toxins (n = 5,576; 34%) (Table 3). Of the nine states in the Brazilian Amazon, Roraima (2°51'50.6"N 60°40'45.2"W) was the one that least used antivenom against spider bites (n = 207; 1.2%) (Table 3). The state also had the highest fatality rate (0.78%) (Table 2). Roraima is located in the extreme North of Brazil and is divided into 18 municipalities.

Three municipalities in Roraima have reference hospitals for accidents caused by spiders: Iracema (2°10'09.1"N 61°03'19.9"W), Amajari (3°39'18.2"N 61° 25'08.6"W) and the capital, São Luiz (1°00'44.6"N 60°02'16.4"W) (Figure 7 A ). The incidence of spider bites by municipality in the Brazilian Amazon is shown in Figure 7 B. Antivenom against spider toxins allocated to reference hospitals in the Amazon can also be administered to patients stung by *Tityus* scorpions. This suggests that the number of vials used in the Brazilian Amazon for the treatment of spider bites may have been lower than that presented in Table 3. According to the Dr. Rosemary Costa Pinto Health Surveillance Foundation of Amazonas [54]. More than 16,000 ampoules of antivenom were distributed in Amazonas in 2022 (Table 3). The quantity of ampoules allocated to and used in in reference hospitals must be recorded in the Strategic Product Information System (SIES).

### Perspectives for the spatial distribution of reference hospitals in the Amazon

The asymmetric distribution of referral hospitals in the Amazon contributes to the risk of death from animal envenoming in remote areas (Figure 7 A ) [13]. The lack of treatment centers and the poor performance of health services are widespread in rural Amazon [55]. In Brazil, antivenoms against toxins from venomous animals are only available in referral hospitals of the Unified Health System (SUS). For most riverside communities, public hospitals represent the only option for treating neglected tropical diseases.

In the Brazilian Amazon, tertiary hospitals are located in the most developed cities in the region, such as Manaus (3°07'48.7"S 60°01'24.7"W) and Belém (1°27'27.2"S 48°29'55.2"W) [41]. Figure 7 A  shows the spatial distribution of hospitals with stocks of antivenom for spider bites in the Brazilian Amazon. In rural Amazon, many reference hospitals have substandard infrastructure in relation to hospitals in capitals [55, 56]. As a specific example, in the municipality of Anori (3°44'53.8"S 61°39'30.8"W), Amazonas, Darlinda Ribeiro Hospital does not have a structure compatible with hospitals in more developed cities such as Santarém (2°26'27.4"S 54°42'59.2"W), Pará, and Araguaína (7°11'37.3"S 48°12'58.5"W), Tocantins. This suggests financial constraints of impoverished municipalities that do not have their own budget to establish new health units [44].

Regional studies provide evidence that many rural hospitals lack specialist doctors and intensive care units for severely envenomed patients [14, 17, 44, 57]. Residents of indigenous and non-indigenous communities attacked by venomous animals are encouraged by local health unit personnel to seek urban hospitals due to the lack of antivenom [58]. Natural barriers, such as rivers and forests, can also delay the initiation of antivenom treatment. It is important to highlight that non-Amazonian regions with a lower lethality rate from spider bites have a higher number of referral hospitals (Figure 7 A) [10, 44, 54].

In the Brazilian Amazon, basic health units (UBSs), where primary health care is provided, have a greater spatial distribution than tertiary hospitals [55]. In the state of Amazonas, there are river-based UBSs able to provide basic health services to communities along the tributaries of the Amazon River. 

Medical boats have refrigeration equipment to maintain medicines that require a specific temperature, such as antivenom. Since many human communities targeted by venomous animals live near riverbanks, reference river UBSs can be strategically positioned on large navigable rivers, such as the Negro, Madeira, Branco, Purus, and Juruá, which serve as access points to several cities with better healthcare options.

Antivenom distribution strategies, as well as the implementation of new health units in the Amazon, should take into account the natural characteristics of the region. Poor road infrastructure and insufficient transportation options are longstanding problems that contribute to the region's geographic isolation. We believe that river-based UBSs are an alternative to increase access to antivenom therapy in the remote Amazon.

Geographic models that mimic patient journeys can be used to estimate the distance between vulnerable communities and Amazonian hospitals [59]. Knowing where the communities most affected by venomous animals are located and what health services are available in a timely manner makes a difference for victims of animal envenoming. Regional policymakers should be informed about alternatives that can increase access to antivenom therapy in the Amazon. Plans to decentralize treatment points in the region depend on coordinated efforts, expanded epidemiological monitoring, and the establishment of health teams in areas far from the capitals.

## Conclusion

The Amazon rainforest is rich in spider species. Three genera of spiders in the Brazilian Amazon are responsible for accidents with clinical repercussions: *Phoneutri*a, *Loxosceles* and *Latrodectus*. Although many residents of rural communities use protective equipment, such as boots and gloves, venomous animals are found inside their homes, where residents usually do not use personal protection.

The number of spider accidents presented in this study is surely underestimated due to the lack of permanent epidemiological monitoring in many rural regions of the Amazon. Our research shows that many reference hospitals in the Amazon are located close to river banks.

In the Brazilian state of Amazonas, batches of antivenom coming from São Paulo are stored in an Immunobiological Distribution Center in the capital, Manaus, and then distributed to municipal health authorities by boat. The regional allocation of antivenoms in reference hospitals is determined according to accident records in the SINAN database.

Reference hospitals in the Amazon are important for impoverished indigenous and non-indigenous communities that depend on medical assistance from the Unified Health System (SUS). Riverside healthcare facilities that provide services along the main rivers of the Amazon region can serve as treatment points for accidents caused by venomous animals. It is important to identify and prioritize areas with a high risk of animal envenoming. It is also necessary to integrate efforts between governments and regional research groups that study local health problems.

### Abbreviations

SIES: Strategic Product Information System; SINAN: Brazilian Notifiable Diseases Information System; SUS: Unified Health System. 

## Availability of data and materials

 All data generated or analyzed during this study are included in this article.
